# Bone marrow biopsy in geriatric patients above the age of 85 years: invaluable or unnecessary? A retrospective analysis

**DOI:** 10.1007/s00277-024-05650-x

**Published:** 2024-02-10

**Authors:** Kailun David Zhang, Edgar Jost, Jens Panse, Reinhild Herwartz, Katharina Lindemann-Docter, Danny Jonigk, Kim Kricheldorf, Anja Köchel, Nicolas Sauerbrunn, Tim H. Brümmendorf, Steffen Koschmieder, Susanne Isfort

**Affiliations:** 1 Department of Hematology, Oncology, Hemostaseology and Stem Cell Transplantation, Faculty of Medicine, RWTH Aachen University, Pauwelsstr. 30, 52074 Aachen, Germany; 2 Center for Integrated Oncology, Aachen Bonn Cologne Düsseldorf (CIO ABCD), Aachen, Germany; 3 Department of Neurology, Kliniken Maria Hilf Mönchengladbach, Mönchengladbach, Germany; 4 German Center for Lung Research (DZL), BREATH, Hannover, Germany; 5 Present Address: Department of Hematology, Hemostasis, Oncology and Stem Cell Transplantation, Medical School Hannover, Hannover, Germany

**Keywords:** Bone marrow biopsy, Geriatric, Hematology, Oncology, Staging, Diagnostics, Follow-up, Survival

## Abstract

Bone marrow biopsy (BMB) is a well-established diagnostic tool for various hematological, oncological, and other medical conditions. However, treatment options for geriatric patients (pts) facing these diseases are often constrained. In this single-center, retrospective analysis we assessed the diagnostic value of BMB in geriatric pts aged ≥ 85 years and examined its impact on therapeutic decisions. We examined 156 BMB procedures in 129 pts, extracting data from the electronic patient records and applying descriptive statistical methods. Nearly half of the primary diagnostic procedures (26; 44.1%) resulted in a modification of the initially suspected diagnosis. Notably, 15 (25.4%) of these procedures, led to changes in both the diagnosis and planned interventional treatment. Among the 15 follow-up procedures (36.6%), disease progression was initially suspected based on symptoms, but BMB results excluded such progression. In lymphoma staging biopsies, only 2 (3.6%) prompted a change in therapeutic intervention. Importantly, no BMB-related complications, such as bleeding, infection or nerve damage, were reported. Median survival after BMB was 16.1 months across all pts, yet it varied based on the diagnosis and comorbidity score. The survival of pts with a change in therapy based on BMB results did not significantly differ from those who did not undergo a therapy change. In conclusion, BMB proved to be generally safe and beneficial in this geriatric cancer patient cohort beyond the age of 85 years. However, the advantages of lymphoma staging in this patient population warrant further consideration.

## Introduction

With rising life expectancy of the general population, physicians are confronted with the decision of whether to perform invasive diagnostic procedures in elderly patients (pts). According to the United Nations, the amount of citizens aged 80 years or older is anticipated to triple by the year 2050, compared to 2019 [1]. Treatment options for geriatric pts with cancer are comparatively limited when contrasted with young adults, as most of the clinical trials assessing new treatment regimens typically restrict participation to younger and fitter pts. However, in recent years, there has been a noteworthy trend towards adapting clinical trials and treatment regimens to cater to elderly pts, subsequently incorporating them into clinical routines [2–12].

In our project, we specifically included pts aged 85 years and beyond, a demographic exceeding the average life expectancy in almost all countries [1].

BMB is an invasive diagnostic procedure that holds a pivotal role in the diagnostic cascade of hematologic malignancies, where bone marrow (BM) involvement often serves as one of the primary diagnostic criteria. It usually involves a two-step approach conducted in a single setting: BM aspiration, where blood derived from the BM is aspirated with a syringe, and a trephine BMB, in which a section of the bone containing the BM is withdrawn to preserve the BM architecture [13]. Generally, BMB is considered a safe procedure with a low rate of complications. A study by Bain and colleagues in 2004 reported an adverse event rate of 0.07% [14], and similar frequencies were observed in prior studies [15–17]. More severe complications may include nerve damage [18, 19], infection [20, 21] and bleeding [22–24], and, in some rare cases, escalate to a life-threatening degree [25, 26]. The acceptability of the risk associated with BMB lies in its potential to provide consequential therapeutic decisions for the patient, irrespective of their age. In our retrospective analysis, our objective was to assess whether geriatric pts undergoing BMB as a routine diagnostic procedure derive substantial benefits from the procedure. We aimed to evaluate the proportion of BMB procedures that significantly contribute to the diagnostic workup and therapeutic decision-making, while also scrutinizing complications and limitations associated with the procedure.

## Methods

We conducted a retrospective analysis of data from pts aged 85 years or older who underwent BMB performed between 2001 and 2020 at RWTH Aachen University Hospital, encompassing both in- and out-patient settings. The data were extracted from the electronic patient records (ePR)). Additional information, including patient history, physical examination details, laboratory assessment, radiological examinations, complications post-BMB (such as bleeding events, bone or local infections, organ damage, and pain) and follow-up data, were also retrieved from the ePR.

To objectively quantify pts’ comorbidity, we employed a modified version of the Cumulative Illness Rating Scale (CIRS) [27]. This scale assigns a score between 0 (no illness) and 4 (severe illness) to each of the 14 organ systems [28]. Commonly used in the evaluation of comorbidity during treatment decisions for pts with chronic lymphocytic leukemia [29], the scoring was retrospectively calculated for each patient at the time of the first BMB using the guidelines presented by Salvi et al. [30]. The main hematological diagnosis of pts was excluded from scoring, following the customary practice for pts with hematologic disorders [31]. In cases where the guidelines did not allow for unambiguous scoring, the lower of two scores was chosen to avoid overrating comorbidity. The Total Score (TSC) represents the sum of scores across all organ systems.

BMBs were analyzed according to the three following categories: “Diagnostic”, “Staging” and “Follow-up”. BMBs were categorized as “Diagnostic” if no hematological disease had been previously diagnosed or if BMB results were pertinent or obligatory for diagnosis. BMBs were categorized as “Staging”, if a malignancy had already been diagnosed through other methods, and BMB was solely performed to complete staging. BMBs were labeled as “Follow-up”, if conducted after initial “diagnostic or staging” BMB. To distinguish BMBs in lymphoma relapse, a BMB was considered “Staging” if the relapse had already been diagnosed via biopsy or radiology, and “Follow-up”, if the relapse was not confirmed at the time of the BMB.

Treatment was classified into interventional and supportive categories. Interventions with antineoplastic potential, including conventional chemotherapy, radiation, tyrosine kinase inhibitors, hydroxyurea and corticosteroids (categorized as interventional, unless explicitly stated as best supportive care in the ePR) were considered.

**Fig. 1:**
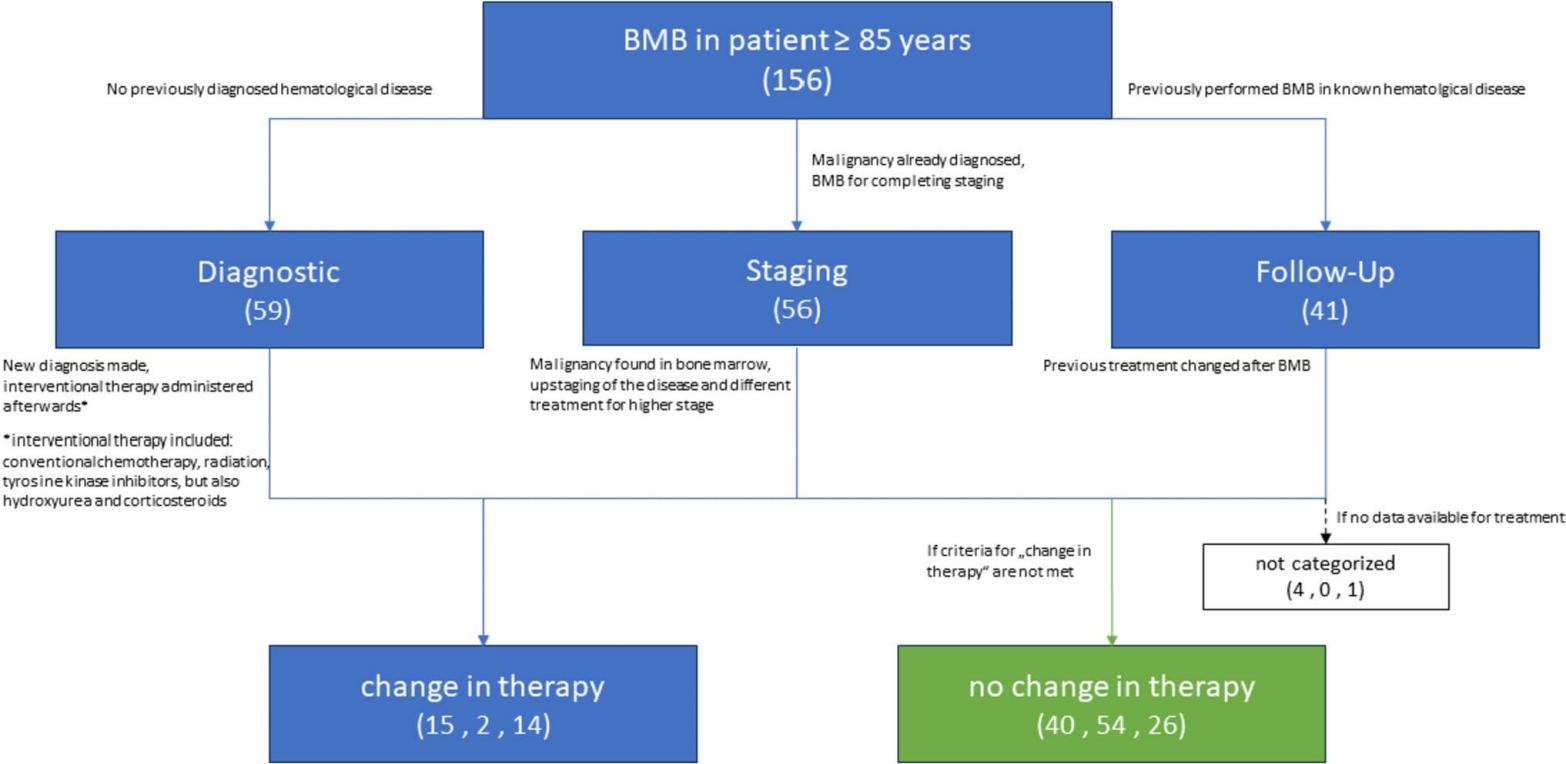
Approach to evaluating the BMB’s influence on therapy. The numbers in brackets represent the number of cases in this study. In the third row, the first number represents the cases from “Diagnostic”, the second number represents the cases from “Staging” and the third number represents the cases from “Follow-Up”

Results of BMBs were categorized based on their potential influence on therapeutic decision- making (“change in therapy”) or lack thereof (“no change in therapy”) (see Fig. 1.). For “Diagnostic” procedures, BMB was deemed to have an impact on therapy if a previously unsuspected diagnosis resulted from the BMB and interventional therapy was consecutively administered. “Staging” procedures were considered influential if BMB revealed infiltration of the diagnosed neoplasm, thereby influencing staging and the chosen treatment regimen. “Follow-up” procedures were considered influential if previously chosen therapy changed due to BMB results. In cases, where no documentation of treatment was available (e.g. if treatment was performed at another center), no categorization was made regarding the influence on therapy.

For pts with multiple BMBs, only the first BMB was considered for survival analyses and evaluations concerning age, comorbidity and general condition.

Statistical analysis involved the unpaired two-sample Student’s t-test for mean values, Spearman’s rank correlation coefficient for intraindividual correlation of two variables, and GraphPad Prism 8 software for survival analyses. Survival analysis and comparison was conducted using the logrank (Mantel-Cox) test, with a significance level set at α = 0.05 for all statistical tests.

The study received approval from the local Ethics Committee (Internal file number EK 309/22).

## Results

### Description of patient cohort

A total of 156 BMBs on 129 individual pts were included in the analysis. Pts characteristics are detailed in Table 1. The mean age was 87.1 ± 2.2 years (range 85–95 years), with 57 (44.1%) being male. The median ECOG Score was ECOG 2 (2, 2), and the mean CIRS TSC was 14.5 ± 5.25. The majority of pts exhibited higher scores for “hypertension” (median score 2; range 2, 3), and “cardiac comorbidities” (median score 2; range 2, 3). Notably, 118 (91.5%) pts scored 2 or higher in three or more organ systems, indicating the prevalence of relevant comorbidities across multiple systems.

**Table 1:** Characteristics of pts 85 years and beyond who received a BMB

Number of pts	129
Number of BMBs	156
Age (years)	
Range	85–95
Median (CI)	87.1 (86.6, 87.6)
Mean ± SD	87.06 ± 2.2
Gender	
Male	57
Female	72
ECOG Score	
0	2
1	38
2	43
3	31
4	12
unknown	3
CIRS score	
Mean Total Score ± SD	14.5 ± 5.3
Mean Severity Index ± SD	1.1 ± 0.4
Mean Comorbidity Index ± SD	4.9 ± 2
Performed BM procedure	
Both cytology and histology	124
Cytology only	29
Histology only	3

### Performed procedures, complications and main diagnoses

Seventeen pts underwent more than one BMB. In 124 out of the 156 procedures (79.5%), both BM aspiration cytology and trephine biopsy were performed (29 BMBs with aspiration cytology report only and 3 procedures with trephine biopsy only). Among these, 105 cases (84.7%) had matching results, while the remaining 19 cases presented methodological issues (7 cases) or discrepant findings (5 cases with pathological findings in either cytology or histology, and 7 of them were resolved through interdisciplinary review).

The most frequent hematologic/oncologic diagnoses included Diffuse Large B-Cell Lymphoma (DLBCL) in 34 cases (21.8%) and acute myeloid leukemia (AML) in 28 cases (17.8%). Notably, no instances of BM infiltration by a solid tumor were observed in our cohort.

Out of the 156 BMBs, 59 (37.8%) were categorized as diagnostic, 56 (35.9%) as staging, and 41 (26.3%) as follow-up.

No adverse effects, such as clinically relevant bleeding, severe pain or nerve damage were reported.

### Diagnostic interventions (59 procedures)

Table 2 outlines BMB indications and resulting diagnoses. In 28 procedures (47.5%), BMBs confirmed the suspected diagnosis, while in 26 cases (44.1%), results altered the initial suspicion. Five cases (8.5%) showed no pathological findings. Approximately half of the diagnostic BMBs (29; 49.2%) resulted in consecutive interventional treatment, while supportive care or no treatment was administered in 26 cases (44.1%).

**Table 2:** Suspected diagnosis before BMB and outcome thereof

Indication/Suspected diagnosis before BMB		Resulting diagnosis upon BMB	
Bicytopenia or pancytopenia of unknown origin	14	Myelodysplastic syndrome	4
		Acute myeloid leukemia	1
		Diffuse large B-cell lymphoma	1
		Bone marrow aplasia	2
		Immune thrombocytopenia	1
		LGL-leukemia	1
		Marginal zone lymphoma	1
		No hematologic diagnosis	2
		Vitamine B12 deficiency	1
Acute leukemia	13	**Acute myeloid leukemia**	10
		Acute lymphoblastic leukemia	1
		Myelodysplastic syndrome	1
		Mantle cell lymphoma	1
Myelodysplastic syndrome	10	**Myelodysplastic syndrome**	4
		**Inconclusive for MDS, clinical diagnosis of MDS**	2
		Myelodysplastic syndrome and indolent lymphoma	1
		Bone marrow tuberculosis	1
		Bone marrow aplasia	1
		Immune thrombocytopenia	1
Multiple myeloma	2	**Multiple myeloma**	1
		Monoclonal gammopathy of unkown significance	1
Chronic myelomonocytic leukemia	3	**Chronic myelomonocytic leukemia**	2
		Acute myeloid leukemia	1
Chronic myeloid leukemia	1	**Chronic myeloid leukemia**	1
(other) Myeloproliferative neoplasm	4	**Myeloproliferative neoplasm**	4
Lymphoplasmacytic lymphoma	1	**Lymphoplasmacytic lymphoma**	1
Chronic lymphocytic leukemia	1	**Chronic lymphocytic leukemia**	1
(suspected paraneoplastic) Autoimmune hemolysis	1	Indolent non-Hodgkin's lymphoma (not further specified)	1
Immune thrombocytopenia	1	**Immune thrombocytopenia**	1
Thrombocytopenia of unknown origin	4	Immune thrombocytopenia	1
		Immune thrombocytopenia secondary to an indolent lymphoma	1
		Myelodysplastic syndrome	1
		No hematologic diagnosis	1
Agranulocytosis	1	Toxic/drug-induced agranulocytosis	1
Eosinophilia	1	No hematologic diagnosis, reactive eosinophilia	1
Systemic mastocytosis	1	No hematologic diagnosis	1
Second biopsy for confirmation of BM tuberculosis	1	**Bone marrow tuberculosis**	1

Highlighted in bold are results from BMBs that match the suspected diagnosis

### Staging interventions (56 procedures)

**Fig. 2:**
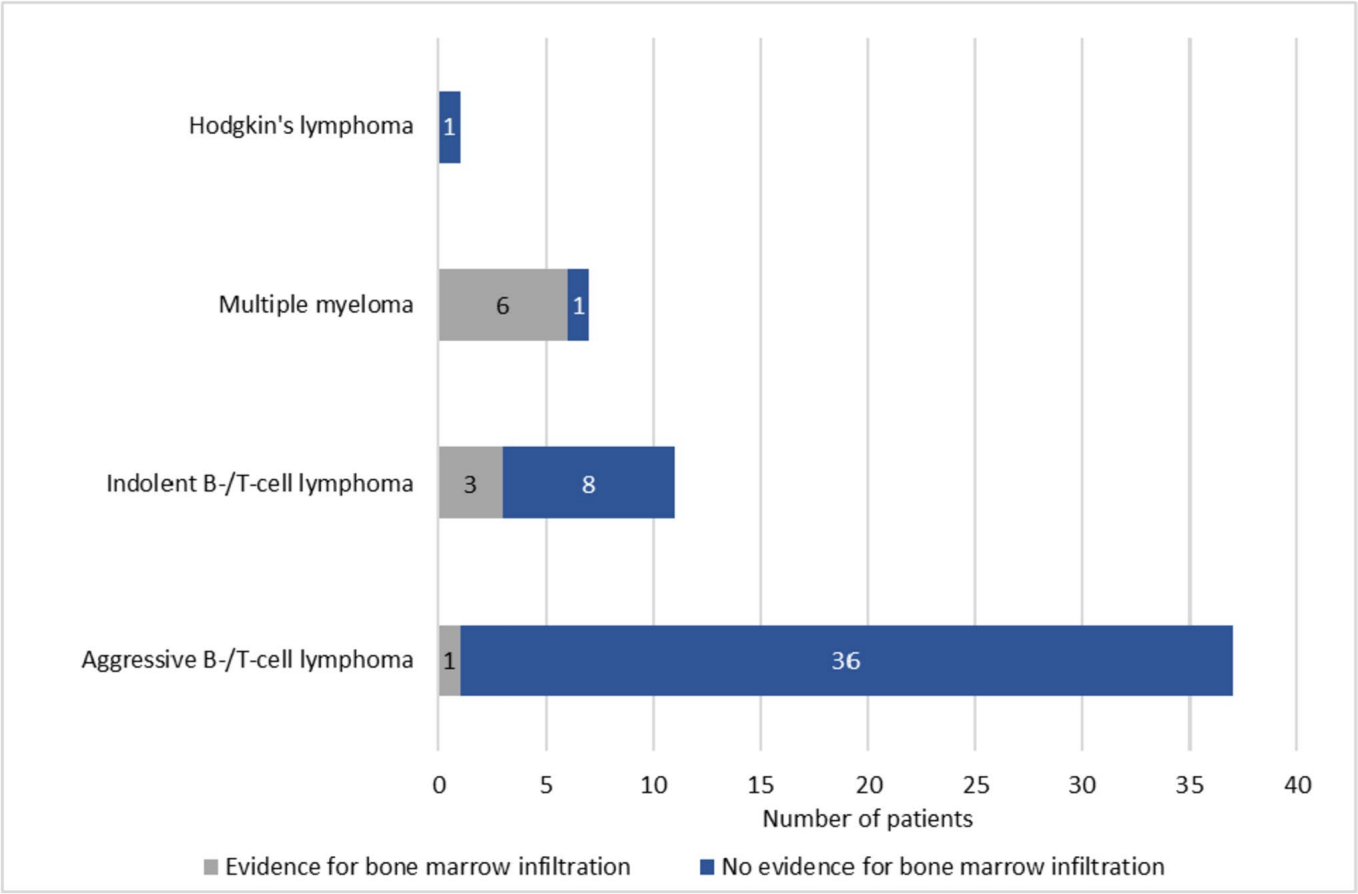
Outcome of 56 BMBs for staging in different types of lymphoma. Among the 37 aggressive B-/T-cell lymphomas, 33 were DLBCL, one was Burkitt’s lymphoma, 3 were not otherwise specified high-grade lymphomas

All “Staging” BMBs were conducted on 54 lymphoma pts, including 8 with lymphoma relapse after previous therapy. Manifestation of the known lymphoma in the BM was detected in 10 staging-biopsies, while 4 led to changes in the stage according to the Ann-Arbor classification. The most common lymphoma type was aggressive B-/T-cell lymphoma in 37 (82.2%) cases, with 33 being DLBCLs. In 36 out of 37 staging BMBs on aggressive B-/T-cell lymphoma, no evidence of BM infiltration was found (see Fig. 2). 2 out of 36 aggressive B-/T-cell lymphoma pts showed bicytopenia or pancytopenia upfront, but BMB revealed no evidence of lymphoma infiltration. Only 2 pts with indolent B-/T-cell lymphoma (3.6%) experienced a change in therapeutic intervention across all staging biopsies.

### Follow-up interventions (41 procedures)

**Fig. 3:**
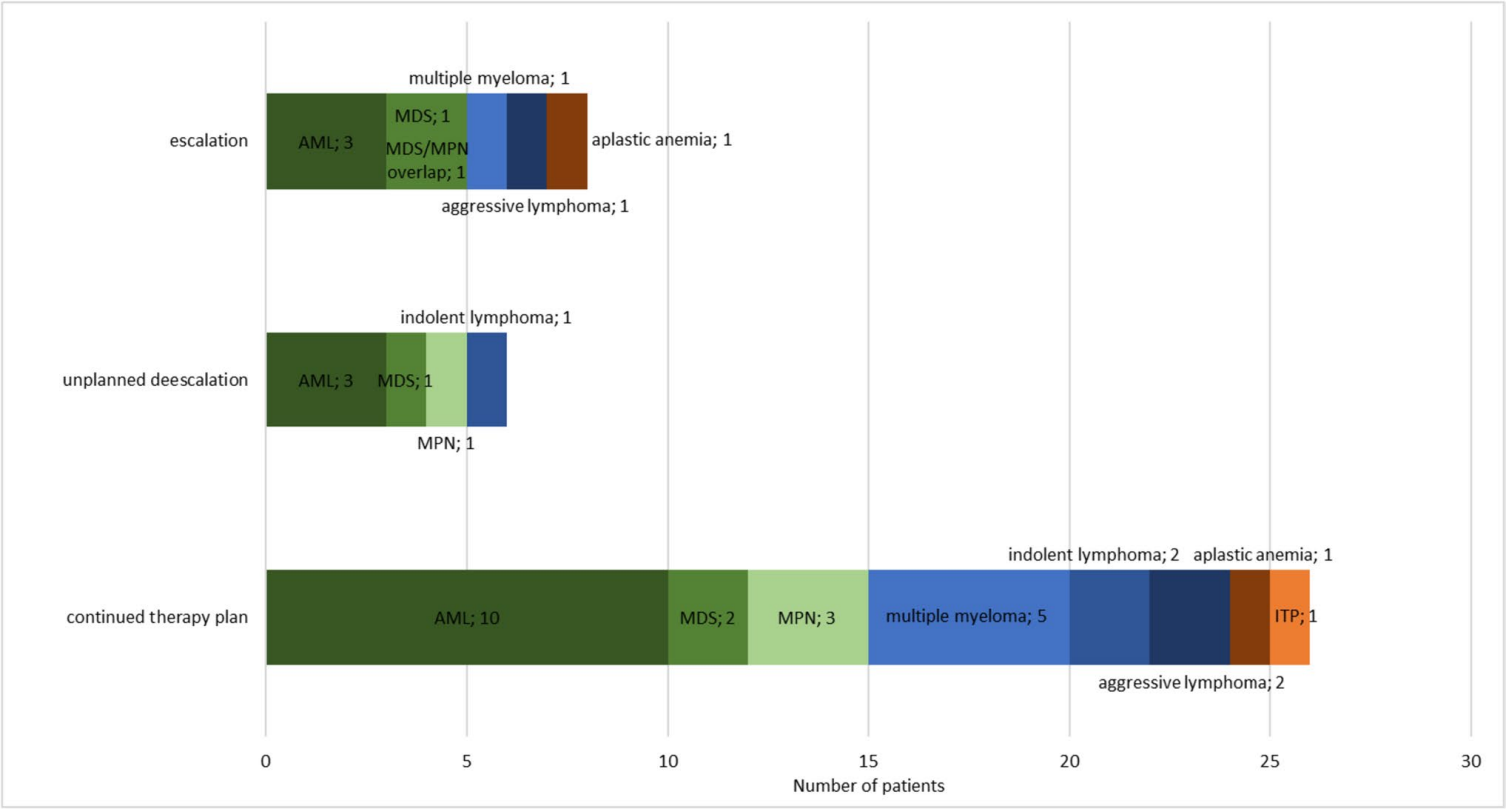
Therapeutic influence of 41 follow-up BMBs; AML, acute myeloid leukemia; MDS, myelodysplastic syndrome; MPN, myeloproliferative neoplasm; ITP, immune thrombocytopenia

“Follow-up” BMBs were performed on 27 pts with various diseases, with acute myeloid leukemia being the most common subgroup (16 cases). Among the procedures, 12 (29.3%) showed a response to treatment, while 15 (36.6%) and 12 (29.3%) revealed stable disease or progressive disease, respectively. One biopsy resulted in diagnosis of a second disease. Among the 12 BMBs resulting in progressive disease, this was previously suspected in 10 cases due to peripheral blood findings or symptoms. In 14 cases (34.1%), treatment was escalated or deescalated after BMB (Fig. 3).

### Survival aspects and influence of comorbidities

**Fig. 4:**
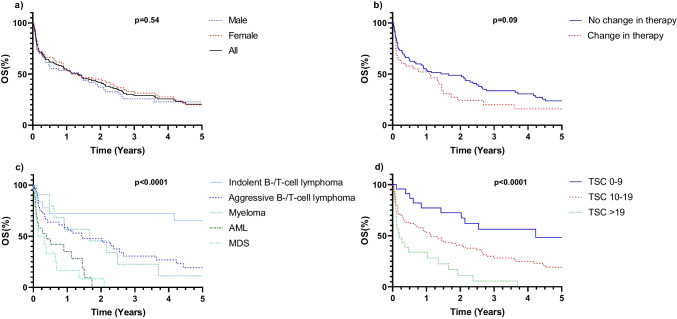
Kaplan–Meier survival curve analysis of overall survival, grouped for change in therapy, main diagnosis and CIRS Total Score; OS, overall survival. **a)** OS of all, male and female pts. *p* = 0.54 between male and female pts. **b)** OS of pts who had a change in therapy due to BMB and who did not. *p* = 0.09 **c)** OS of pts with the 5 most frequent diagnoses: aggressive B-/T-cell lymphoma, indolent B-/T-cell lymphoma, multiple myeloma, myelodysplastic syndrome, acute myeloid leukemia. *p* < 0.0001 **d)** OS of pts with CIRS Total Score > 19, 10–19 and 0–9. *p* < 0.0001

The median survival in the entire cohort was 16.1 (8.5, 24.6) months, with no significant difference between male and female pts (see Fig. 4a). Pts with a therapy change based on BMB results showed a trend towards worse survival compared to those without therapy changes (see Fig. 4b). Kaplan–Meier curves on survival for the five most frequent diseases are presented in Fig. 4c.

The average CIRS indices in our cohort were as follows: mean TSC was 14.72 ± 5.17. While average CIRS indices between pts with or without a change in therapy due to BMB results were not significantly different, there was a significant correlation between CIRS indices and days until death/last time of information after BMB (Spearman r = 0.35 for TSC, *p* < 0.0001). Pts with a CIRS TSC ≥ 20 had a shorter median survival of 2.3 (1.3, 12.4) months compared to pts with a TSC of 10–19 (median survival of 13.7 (8.1, 25.6) months) or with a TSC of 0–9 (median survival of 50.8 (17.1, 91.1) months) (Fig. 4d), emphasizing the influence of comorbidities on survival in this cohort.

## Discussion

### Patient cohort, complications, main diagnoses, comorbidities and survival aspects

The demographics of an aging society in Western countries underscore the potential future relevance of our analysis. Notably, this study observed no major complications related to BMB, aligning with the very low risk reported in the literature [14–17]. While acknowledging the retrospective nature of our analysis, which may have led to the potential under-detection of non-severe complications like minor or moderate pain, our results affirm that BMB is feasible in a geriatric patient population.

Our patient cohort exhibited a substantial burden of comorbidities, with over 4 out of 5 pts suffering from heart disease or systemic arterial hypertension. Surprisingly, there was no significant correlation between CIRS TSC burden and pts receiving less intensive therapy. However, pts with higher CIRS TSC burden demonstrated significantly shorter survival compared to those with lower CIRS TSC. Additionally, pts with a therapy switch based on BMB results showed a trend towards worse survival, potentially linked disease progression or relapse.

Comparing the survival of our cohort to the general population of similar age (with life expectancy for 85-year old individuals in Germany ranging between 5.18 years for men [32], 6.00 years for women in 2000 [33], and 5.54 years for men [32], and 6.54 years for women [33] in 2020, while the median survival in this analysis was 16.1 months), highlighted a severely reduced life expectancy, likely attributed to malignant diseases. However, pts with indolent B-/T-cell lymphoma, with a median age of 88 years at the time of BMB, exhibited a median survival of over 6 years.

### Diagnostic interventions

In this cohort, BMB results altered the initially suspected diagnosis in half of the pts without prior history of hematological disease. Given that BMB confirmation is a crucial diagnostic criterion in various diseases [34–37], especially myeloid neoplasms, performing this procedure remains essential to align with international standards. A real-world analysis of Myeloproliferative Neoplasm (MPN) pts in Germany revealed that almost 40% of pts with suspected essential thrombocythemia did not undergo upfront BMB to secure the diagnosis [38]. After diagnosis, only half of pts received interventional treatment. Pts receiving only supportive treatment did so either due to lack of physical fitness or because it was the current recommended treatment for their disease (e.g. in myelodysplastic syndrome).

BMB revealed pathological findings in over 90% of cases in our cohort, differing significantly from the study by Manion et al. from 2008 [39]. In their analysis, only 43% of primary diagnostic BMBs yielded a specific diagnosis and several BMBs were conducted due to anemia of unknown origin. Furthermore, only minimal therapeutic success was reported in their pts cohort, which led the authors to the conclusion that a higher threshold for BMBs may be indicated. Differences in biopsy indications between the cohorts suggest that patient populations with a higher threshold for BMB indication, such as ours, may indeed benefit from the procedure. This was supported by the considerable influence of BMB on patients' outcomes in our analysis.

### Staging interventions

For the majority of this cohort, mostly diagnosed with DLBCL, BMB did not reveal BM infiltration. Only in 2 pts with indolent B-/T-cell lymphoma did the Ann-Arbor stage and consecutive therapy change based on BMB results (change in therapeutic intervention), with both pts not showing cytopenia in the peripheral blood upfront.

BMB detects BM involvement in about 14% of DLBCL cases of all ages [40, 41]. In our analysis, the rate for detecting BM involvement in B-/T-cell lymphoma, mostly DLBCL, was much lower at 2.7%. This suggests a lower rate of BM involvement of DLBCL in geriatric pts. A higher Ann-Arbor staging category leads to a different therapeutic regimen for younger pts [42]. In the elderly, attenuated immunochemotherapy regimens are given in all stages [3, 43], and, therefore, BMB has limited therapeutic implications in this setting. For Hodgkin’s lymphoma, PET-CT is recommended for all pts as an alternative staging method to BMB, and routine BMB in PET-negative cases is already obsolete [44, 45].

### Follow-up interventions

In the “Follow-up” cohort, about one-third of pts had their treatment adjusted based on BMB results, either due to disease progression or response to therapy. In most cases with confirmation of disease progression via BMB, there were clinical or laboratory findings suggesting progression before BMB. However, in more than half of the BMBs resulting in no disease progression, peripheral blood counts and clinical status had led to suspicion of a progression upfront. Therefore, symptoms and peripheral blood findings do not seem to be reliable predictors of disease progression in our cohort, emphasizing the impact of BMB for exclusion or confirmation of disease progression.

### Limitations

The retrospective data capture may have introduced bias due to missing information on some pts. This is a single-center experience resulted in a limited patient cohort. Unfortunately, a comparable control group of pts who did not undergo BMB was not available due to the study design and the low frequency of pts beyond 85 years undergoing BMB. Furthermore, due to our focus on hematological malignancies, cases of bone marrow infiltration by solid tumors may have been underrepresented in our analysis.

## Conclusion

In conclusion, BMB seems to be safe in geriatric pts. Despite a high burden of comorbidities and advanced age, interventional therapy is possible for a substantial number of pts, potentially even more so with upcoming targeted treatments like tyrosine-kinase-inhibitors and monoclonal antibodies. For diagnostic purposes, BMB contributed significantly to the diagnostic process and often corrected the initially suspected diagnoses. For confirmation of disease progression, BMB was indispensable, as clinical features alone were much less reliable. For sole staging purposes in lymphoma, BMB had limited therapeutic implications. Comorbidity burden (represented by TSC score) correlated with impaired survival.

## Data Availability

As data was taken retrospectively from our hospital´s electronic patient case files with no further informed consent for data sharing, full unaggregated data set cannot be made available for public use.

## Abbreviations

BMB: Bone marrow biopsy
BM: Bone marrow
Pts: Patients
CIRS: Cumulative Illness Rating Scale; TSC: Total Score
DLBCL: Diffuse large B-cell lymphoma
ECOG: Eastern Cooperative Oncology GroupAML: acute myeloid leukemia
PET-CT: Positron emission tomography-computed tomography

## References

[1] United Nations, Department of Economic and Social Affairs (2020) World population ageing 2019. 10.18356/6a8968ef-en

[2] Seymour MT, Thompson LC, Wasan HS, Middleton G, Brewster AE, Shepherd SF, O’Mahony MS, Maughan TS, Parmar M, Langley RE, Investigators F, National Cancer Research Institute Colorectal Cancer Clinical Studies G (2011). Chemotherapy options in elderly and frail patients with metastatic colorectal cancer (MRC FOCUS2): an open-label, randomised factorial trial. Lancet.

[3] Peyrade F, Jardin F, Thieblemont C, Thyss A, Emile JF, Castaigne S, Coiffier B, Haioun C, Bologna S, Fitoussi O, Lepeu G, Fruchart C, Bordessoule D, Blanc M, Delarue R, Janvier M, Salles B, Andre M, Fournier M, Gaulard P, Tilly H, Grouped’Etude des Lymphomes de l’Adultei (2011). Attenuated immunochemotherapy regimen (R-miniCHOP) in elderly patients older than 80 years with diffuse large B-cell lymphoma: a multicentre, single-arm, phase 2 trial. Lancet Oncol.

[4] Saiz-Rodriguez M, Labrador J, Cuevas B, Martinez-Cuadron D, Campuzano V, Alcaraz R, Cano I, Sanz MA, Montesinos P (2021) Use of Azacitidine or Decitabine for the Up-Front Setting in Acute Myeloid Leukaemia: A Systematic Review and Meta-Analysis. Cancers (Basel) 13(22). 10.3390/cancers13225677 10.3390/cancers13225677 PMC8616518 34830832

[5] Saito Z, Fujita K, Okamura M, Ito T, Yamamoto Y, Kanai O, Hashimoto M, Nakatani K, Sawai S, Mio T (2021). Efficacy and safety of immune checkpoint inhibitors in patients with non-small cell lung cancer aged 80 years or older. Cancer Rep (Hoboken).

[6] Oberic L, Peyrade F, Puyade M, Bonnet C, Dartigues-Cuilleres P, Fabiani B, Ruminy P, Maisonneuve H, Abraham J, Thieblemont C, Feugier P, Salles G, Bijou F, Pica GM, Damaj G, Haioun C, Casasnovas RO, Farhat H, Le Calloch R, Waultier-Rascalou A, Malak S, Paget J, Gat E, Tilly H, Jardin F (2021). Subcutaneous Rituximab-MiniCHOP Compared With Subcutaneous Rituximab-MiniCHOP Plus Lenalidomide in Diffuse Large B-Cell Lymphoma for Patients Age 80 Years or Older. J Clin Oncol.

[7] Nebhan CA, Cortellini A, Ma W, Ganta T, Song H, Ye F, Irlmeier R, Debnath N, Saeed A, Radford M, Alahmadi A, Diamond A, Hoimes C, Ramaiya N, Presley CJ, Owen DH, Abou Alaiwi S, Nassar A, Ricciuti B, Lamberti G, Bersanelli M, Casartelli C, Buti S, Marchetti P, Giusti R, Filetti M, Vanella V, Mallardo D, Macherla S, Sussman TA, Botticelli A, Galetta D, Catino A, Pizzutilo P, Genova C, Dal Bello MG, Kalofonou F, Daniels E, Ascierto PA, Pinato DJ, Choueiri TK, Johnson DB, Marron TU, Wang Y, Naqash AR (2021). Clinical outcomes and toxic effects of single-agent immune checkpoint inhibitors among patients aged 80 years or older with cancer: A multicenter international cohort study. JAMA Oncol.

[8] Merli F, Cavallo F, Salvi F, Tucci A, Musuraca G, Nassi L, Merli M, Tani M, Gini G, Ferrari A, Molinari AL, Liberati AM, Conconi A, Matteucci P, Bari A, Scalone R, Ferrero S, Zanni M, Mammi C, Luminari S (2020). Obinutuzumab and miniCHOP for unfit patients with diffuse large B-cell lymphoma. A phase II study by Fondazione Italiana Linfomi. J Geriatr Oncol.

[9] Leroy V, Gerard E, Dutriaux C, Prey S, Gey A, Mertens C, Beylot-Barry M, Pham-Ledard A (2019). Adverse events need for hospitalization and systemic immunosuppression in very elderly patients (over 80 years) treated with ipilimumab for metastatic melanoma. Cancer Immunol Immunother.

[10] Peyrade F, Bologna S, Delwail V, Emile JF, Pascal L, Ferme C, Schiano JM, Coiffier B, Corront B, Farhat H, Fruchart C, Ghesquieres H, Macro M, Tilly H, Choufi B, Delarue R, Fitoussi O, Gabarre J, Haioun C, Jardin F (2017). Combination of ofatumumab and reduced-dose CHOP for diffuse large B-cell lymphomas in patients aged 80 years or older: an open-label, multicentre, single-arm, phase 2 trial from the LYSA group. Lancet Haematol.

[11] Price TJ, Zannino D, Wilson K, Simes RJ, Cassidy J, Van Hazel GA, Robinson BA, Broad A, Ganju V, Ackland SP, Tebbutt NC (2012). Bevacizumab is equally effective and no more toxic in elderly patients with advanced colorectal cancer: a subgroup analysis from the AGITG MAX trial: an international randomised controlled trial of Capecitabine. Bevacizumab Mitomycin C Ann Oncol.

[12] Krug U, Koschmieder A, Schwammbach D, Gerss J, Tidow N, Steffen B, Bug G, Brandts CH, Schaich M, Rollig C, Thiede C, Noppeney R, Stelljes M, Buchner T, Koschmieder S, Duhrsen U, Serve H, Ehninger G, Berdel WE, Muller-Tidow C (2012). Feasibility of azacitidine added to standard chemotherapy in older patients with acute myeloid leukemia–a randomised SAL pilot study. PLoS One.

[13] Le Clef Q, Menter T, Tzankov A (2019). Our approach to bone marrow biopsies in cytopenia. Pathol Res Pract.

[14] Bain BJ (2006). Morbidity associated with bone marrow aspiration and trephine biopsy - a review of UK data for 2004. Haematologica.

[15] Bain BJ (2005). Bone marrow biopsy morbidity: review of 2003. J Clin Pathol.

[16] Bain BJ (2004). Bone marrow biopsy morbidity and mortality: 2002 data. Clin Lab Haematol.

[17] Bain BJ (2003). Bone marrow biopsy morbidity and mortality. Br J Haematol.

[18] McGoldrick NP, Green C, Connolly P (2012). Gluteal compartment syndrome following bone marrow biopsy: a case report. Acta Orthop Belg.

[19] Lowenthal RM, Taylor BV, Jones R, Beasley A (2006). Severe persistent sciatic pain and weakness due to a gluteal artery pseudoaneurysm as a complication of bone marrow biopsy. J Clin Neurosci.

[20] Khakwani M, Srinath S, Pratt G, Moss P (2019). A rare complication of bone marrow aspiration and trephine biopsy: Staphylococcus aureus osteomyelitis and septicaemia. Br J Haematol.

[21] Morotti A, Barozzino MC, Guerrasio A (2016). rare bone marrow biopsy complication: A challenging case of Sacroiliitis and Staphilococcus Aureus Sepsis. Clin Pract.

[22] Houtman D, Barten DG, Laurent-de Gast AN (2017). A woman with inguinal pain after a bone marrow biopsy. Ned Tijdschr Geneeskd.

[23] Berber I, Erkurt MA, Kuku I, Kaya E, Kutlu R, Koroglu M, Yigit A, Unlu S (2014). An unexpected complication of bone marrow aspiration and trephine biopsy: arteriovenous fistula. Med Princ Pract.

[24] Sarigianni M, Vlachaki E, Chissan S, Klonizakis F, Vetsiou E, Anastasiadou KI, Ioannidou-Papagiannaki E, Klonizakis I (2011). Haematoma caused by bone marrow aspiration and trephine biopsy. Hematol Rep.

[25] Al Zahrani Y, Peck D (2016). Median sacral artery injury following a bone marrow biopsy successfully treated with selective trans-arterial embolization: a case report. J Med Case Rep.

[26] Wan Jamaludin WF, Mohamed Mukari SA, Abdul Wahid SF (2013). Retroperitoneal hemorrhage associated with bone marrow trephine biopsy. Am J Case Rep.

[27] Mistry R, Gokhman I, Bastani R, Gould R, Jimenez E, Maxwell A, McDermott C, Rosansky J, Van Stone W, Jarvik L, Group UC (2004). Measuring medical burden using CIRS in older veterans enrolled in UPBEAT, a psychogeriatric treatment program: a pilot study. J Gerontol A Biol Sci Med Sci.

[28] Linn BS, Linn MW, Gurel L (1968). Cumulative illness rating scale. J Am Geriatr Soc.

[29] Goede V, Fischer K, Busch R, Engelke A, Eichhorst B, Wendtner CM, Chagorova T, de la Serna J, Dilhuydy MS, Illmer T, Opat S, Owen CJ, Samoylova O, Kreuzer KA, Stilgenbauer S, Dohner H, Langerak AW, Ritgen M, Kneba M, Asikanius E, Humphrey K, Wenger M, Hallek M (2014). Obinutuzumab plus chlorambucil in patients with CLL and coexisting conditions. N Engl J Med.

[30] Salvi F, Miller MD, Grilli A, Giorgi R, Towers AL, Morichi V, Spazzafumo L, Mancinelli L, Espinosa E, Rappelli A, Dessi-Fulgheri P (2008). A manual of guidelines to score the modified cumulative illness rating scale and its validation in acute hospitalized elderly patients. J Am Geriatr Soc.

[31] Gordon MJ, Churnetski M, Alqahtani H, Rivera X, Kittai A, Amrock SM, James S, Hoff S, Manda S, Spurgeon SE, Choi M, Cohen JB, Persky D, Danilov AV (2018). Comorbidities predict inferior outcomes in chronic lymphocytic leukemia treated with ibrutinib. Cancer.

[32] Federal Statistical Office of Germany, Robert-Koch Institute: Gesundheitsberichtserstattung des Bundes. 2002 and 2022. https://www.gbe-bund.de/gbe/!pkg_olap_tables.prc_set_page?p_uid=gast&p_aid=69135500&p_sprache=D&p_help=2&p_indnr=524&p_ansnr=27031407&p_version=6&D.001=1000001&D.003=42

[33] Federal Statistical Office of Germany, Robert-Koch Institute: Gesundheitsberichtserstattung des Bundes. 2002 and 2022. https://www.gbe-bund.de/gbe/!pkg_olap_tables.prc_set_page?p_uid=gast&p_aid=69135500&p_sprache=D&p_help=2&p_indnr=524&p_ansnr=27031407&p_version=7&D.001=1000001&D.003=43

[34] Tefferi A (2021). Primary myelofibrosis: 2021 update on diagnosis, risk-stratification and management. Am J Hematol.

[35] Killick SB, Bown N, Cavenagh J, Dokal I, Foukaneli T, Hill A, Hillmen P, Ireland R, Kulasekararaj A, Mufti G, Snowden JA, Samarasinghe S, Wood A, Marsh JC, British Society for Standards in H (2016). Guidelines for the diagnosis and management of adult aplastic anaemia. Br J Haematol.

[36] Arber DA, Orazi A, Hasserjian R, Thiele J, Borowitz MJ, Le Beau MM, Bloomfield CD, Cazzola M, Vardiman JW (2016). The 2016 revision to the World Health Organization classification of myeloid neoplasms and acute leukemia. Blood.

[37] Greenberg PL, Tuechler H, Schanz J, Sanz G, Garcia-Manero G, Sole F, Bennett JM, Bowen D, Fenaux P, Dreyfus F, Kantarjian H, Kuendgen A, Levis A, Malcovati L, Cazzola M, Cermak J, Fonatsch C, Le Beau MM, Slovak ML, Krieger O, Luebbert M, Maciejewski J, Magalhaes SM, Miyazaki Y, Pfeilstocker M, Sekeres M, Sperr WR, Stauder R, Tauro S, Valent P, Vallespi T, van de Loosdrecht AA, Germing U, Haase D (2012). Revised international prognostic scoring system for myelodysplastic syndromes. Blood.

[38] Schmidt A, Bernhardt C, Burkle D, Fries S, Hannig CV, Jentsch-Ullrich K, Josting A, Kreher S, Reiser M, Steinmetz HT, Tesch H, Terner S, Schulte A, Crodel CC, Palandri F, Heidel FH (2023). Diagnosis and treatment of MPN in real life: exploratory and retrospective chart review including 960 MPN patients diagnosed with ET or MF in Germany. J Cancer Res Clin Oncol.

[39] Manion EM, Rosenthal NS (2008). Bone marrow biopsies in patients 85 years or older. Am J Clin Pathol.

[40] Han EJ, O JH, Yoon H, Ha S, Yoo IR, Min JW, Choi JI, Choi BO, Park G, Lee HH, Jeon YW, Min GJ, Cho SG (2022) Comparison of FDG PET/CT and Bone Marrow Biopsy Results in Patients with Diffuse Large B Cell Lymphoma with Subgroup Analysis of PET Radiomics. Diagnostics (Basel) 12(1). 10.3390/diagnostics12010222 10.3390/diagnostics12010222 PMC8774933 35054389

[41] Vishnu P, Wingerson A, Lee M, Mandelson MT, Aboulafia DM (2017). Utility of bone marrow biopsy and aspirate for staging of diffuse large b cell lymphoma in the era of positron emission tomography with 2-Deoxy-2-[Fluorine-18]fluoro-deoxyglucose integrated with computed tomography. Clin Lymphoma Myeloma Leuk.

[42] Tilly H, Morschhauser F, Sehn LH, Friedberg JW, Trneny M, Sharman JP, Herbaux C, Burke JM, Matasar M, Rai S, Izutsu K, Mehta-Shah N, Oberic L, Chauchet A, Jurczak W, Song Y, Greil R, Mykhalska L, Bergua-Burgues JM, Cheung MC, Pinto A, Shin HJ, Hapgood G, Munhoz E, Abrisqueta P, Gau JP, Hirata J, Jiang Y, Yan M, Lee C, Flowers CR, Salles G (2022). Polatuzumab vedotin in previously untreated diffuse large B-Cell Lymphoma. N Engl J Med.

[43] Weidmann E, Neumann A, Fauth F, Atmaca A, Al-Batran SE, Pauligk C, Jager E (2011). Phase II study of bendamustine in combination with rituximab as first-line treatment in patients 80 years or older with aggressive B-cell lymphomas. Ann Oncol.

[44] Hamilton R, Andrews I, McKay P, Leach M (2014). Loss of utility of bone marrow biopsy as a staging evaluation for Hodgkin lymphoma in the positron emission tomography-computed tomography era: a West of Scotland study. Leuk Lymphoma.

[45] El-Galaly TC, d'Amore F, Mylam KJ, de Nully BP, Bogsted M, Bukh A, Specht L, Loft A, Iyer V, Hjorthaug K, Nielsen AL, Christiansen I, Madsen C, Johnsen HE, Hutchings M (2012). Routine bone marrow biopsy has little or no therapeutic consequence for positron emission tomography/computed tomography-staged treatment-naive patients with Hodgkin lymphoma. J Clin Oncol.

